# Phase angle and World Health Organization criteria for the assessment of nutritional status in children with osteogenesis imperfecta

**DOI:** 10.1016/j.rppede.2016.03.010

**Published:** 2016

**Authors:** Vicky Nogueira Pileggi, Antonio Rodolpho Hakime Scalize, José Simon Camelo

**Affiliations:** Faculdade de Medicina de Ribeirão Preto, Universidade de São Paulo (FMRP-USP), Ribeirão Preto, SP, Brazil

**Keywords:** Osteogenesis imperfecta, Phase angle, Nutritional status

## Abstract

**Objective:**

To compare the phase angle of patients with osteogenesis imperfecta treated at a tertiary university hospital with patients in a control group of healthy children, and to assess the nutritional status of these patients through the body mass index proposed by the World Health Organization.

**Methods:**

Cross-sectional study carried out in a university hospital that included seven patients with osteogenesis imperfecta and a control group of 17 healthy children of the same gender and age. Weight and height were measured and bioelectrical impedance was performed. Subsequently, the phase angle was calculated based on resistance and reactance values.

**Results:**

The phase angle of the group of children with osteogenesis imperfecta was significantly lower than that of the control group (*p*<0.05). The body mass index criterion for age of the World Health Organization showed no difference between groups.

**Conclusions:**

Children with osteogenesis imperfecta have a nutritional risk detected by the phase angle, which is a useful tool for nutritional screening. The calculation result could help in the diet therapy of patients with osteogenesis imperfecta.

## Resumo

**Objetivo:**

Comparar o ângulo de fase de pacientes com osteogênese imperfeita atendidos em um hospital universitário terciário com pacientes de um grupo controle de crianças saudáveis, bem como avaliar o estado nutricional desses pacientes pelo índice de massa corporal proposto pela Organização Mundial de Saúde.

**Métodos:**

Estudo transversal feito em hospital universitário que incluiu sete pacientes com osteogênese imperfeita e um grupo controle composto por 17 crianças saudáveis de mesmo sexo e idade. Foram aferidos peso e estatura e foi feito o exame de impedância bioelétrica. Posteriormente, o ângulo de fase foi calculado a partir dos valores de resistência e reactância.

**Resultados:**

O ângulo de fase do grupo de crianças com osteogênese imperfeita foi significativamente menor do que o do grupo controle (*p*<0,05). O critério de índice de massa corporal por idade da Organização Mundial de Saúde não mostrou diferença entre os grupos.

**Conclusões:**

Crianças com osteogênese imperfeita têm um risco nutricional detectado pelo ângulo de fase, é uma ferramenta útil para triagem nutricional. O resultado do cálculo poderia auxiliar a dietoterapia de pacientes com osteogênese imperfeita.

## Introduction

Osteogenesis imperfecta (OI) is an inherited disease characterized by bone fragility and predisposition to fractures that occur with minimal or even in the absence of trauma. Patients with this disease often have low bone mass, but studies on the nutritional status of children with OI are scarce.^1^

In 2012, a study of patients with OI types I and III indicated the need for knowledge of body composition, as it is associated with fracture onset.^2^ The authors indicated the need to individualize the diet of individuals with OI to achieve body composition improvement.

The phase angle (PA), obtained from the secondary analysis of bioelectric impedance for analyzing body composition without the use of anthropometric parameters could be used in patients with osteogenesis imperfecta, as anthropometry, especially height measurement, is difficult to perform accurately.^3^^,^ 4 The PA is the arctangent of resistance and reactance ratio (Xc/R), that is, it derived from the bioelectrical impedance evaluation with the use of direct measurements of the components of the R and Xc vector; when used as nutritional status indicator and body cell mass (BCM), it considerably eliminates the errors of analysis by bioelectrical impedance.^5^ Its use has been recommended as an indicator of prognosis in clinical practice. In adults, positive associations have been found between PA and survival of HIV-positive patients,^6^^,^ 7 and those with pulmonary cancer,^8^ as well as sepsis and those undergoing hemodialysis.^9^

In this context, the objective of this study was to compare the PA of patients with osteogenesis imperfecta treated at Hospital das Clínicas of Faculdade de Medicina de Ribeirão Preto of Universidade de São Paulo (HCFRMP-USP) with a control group of healthy children and to assess the nutritional status of these patients using the World Health Organization (WHO) parameters of body mass index for age (BMI/A).

## Method

This was a secondary analysis of a cross-sectional study that included seven children diagnosed with osteogenesis imperfecta (types I, III and IV) treated with pamidronate in HCFMRP-USP. All patients were followed at the Pediatric Endocrinology and Orthopedic Outpatient Clinic at the same hospital. These children do not represent all pediatric patients with OI treated at the hospital. They were randomly assigned to another study and were analyzed separately due to the disease complexity. Data from these patients were compared with those from 17 healthy children of the same gender and age, which comprised the control group. The children from the control group attend the Child Care and Pediatric Outpatient Clinic of Cuiabá Health Unit in the municipality of Ribeirão Preto. This location was chosen as it cares for healthy children of the same socioeconomic level of those treated at the study tertiary hospital. They were stratified by gender and age. There was no pairing, as they are independent groups.

Four children from the study group and 12 from the control group signed the term of consent (aged>7 years); all patients had the informed consent form signed by their parents/tutors. This was a secondary analysis of the study on the prevalence of malnutrition in HCFMRP-USP carried out in 2013.^9^ The study was approved by Institutional Review Board of HCFMRP-USP.

Data on weight and bioelectrical impedance were collected according to international procedures.^10^ The height/length was measured, in some cases, on the patients' own bed, as they were unable to remain in the standing position (*n*=6). Body mass index (BMI) was calculated for BMI/A classification according to the WHO charts.^11^

The PA was calculated according to the formula: *Φ*=(*Xc*/*R*)×(180º/π), in which *Xc* is the reactance value, *R* is the resistance value and pi is the mathematical value of 3.1415. This conversion is performed to convert the final value from radians into degrees.^12^

For the statistical analysis of data, the following software were used: SPSS 20.0 (Statistical Package for the Social Sciences, Westlands Road, Quarry Bay, Hong Kong, 2009) and R (R Foundation for Statistical Computing, University of Auckland, New Zealand, 1993). Nonparametric statistics were used, considering that there is no certainty of normal distribution for the anthropometric data of patients with OI. The Mann-Whitney test for independent samples was used to compare the variables of interest.

## Results

Anthropometrics and body composition analysis by bioelectrical impedance, as well as the phase angle calculations are shown in Table 1.

**Table 1:** Sample characteristics: demographic data of the groups.

	Osteogenesis (*n*=7)						Control (*n*=17)					*p*-value^a^
	Mean	Median	SD^b^	Minimum	Maximum		Mean	Median	SD^b^	Minimum	Maximum	
Age (months)	104.7	123	52.42	10	158		116.1	122	39.81	10	160	0.901
Weight (kg)	24.94	16.5	20.33	6.86	68.65		33.03	31.7	10.76	10.01	48.3	0.047
Height (m)	1.06	10.8	0.26	0.65	1.51		1.37	1.4	0.2	0.78	1.67	0.011
BMI (kg/m^2^)	19.31	18.59	5.34	13.25	30.11		16.83	16.66	1.58	14.19	20.08	0.187
Resistance	690.6	706.3	158.3	503	965		728.6	726	110.4	503	964.33	0.455
Reactance	56.71	51	15.39	35	76		69.49	70.66	9.36	52	86	0.055
Phase angle	4.74	4.8	0.93	3.58	5.95		5.5	5.48	0.57	4.65	6.79	0.047

a Mann-Whitney *U* test.

b SD=Standard deviation.

**Figure 1:**
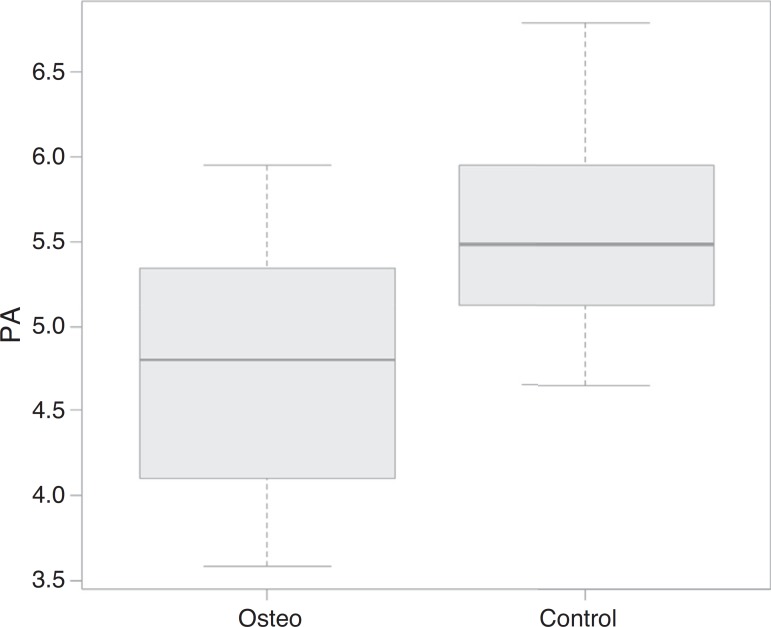
Comparison of the phase angle (PA) data between the groups of patients with osteogenesis imperfecta (=osteo) and children in the control group (=control).

According to the Mann-Whitney test, patients with osteogenesis had a significantly lower classification of weight, height and phase angle when compared to that of the control group. The data distribution regarding the phase angle can be observed in Fig. 1.

All patients from the control group were classified as having normal weight according to the WHO criteria. In the group of patients with osteogenesis, two were classified as obese (BMI percentile>97) and the others as having normal weight (Table 2).

**Table 2:** Nutritional status classification according to the WHO criteria and BMI/A.

Group	Patient	Gender	Age (months)	BMI (kg/m^2^)	Nutritional classification
O	1	Female	79	13.25	Normal weight
O	2	Male	158	30.11	Obesity
O	3	Female	10	16.24	Normal weight
O	4	Female	123	16.84	Normal weight
O	5	Male	79	20.83	Obesity
O	6	Female	131	18.59	Normal weight
O	7	Female	153	19.36	Normal weight
C	8	Male	10	16.45	Normal weight
C	9	Male	78	15.48	Normal weight
C	10	Male	78	15.52	Normal weight
C	11	Male	78	16.73	Normal weight
C	12	Male	131	15.72	Normal weight
C	13	Male	150	17.60	Normal weight
C	14	Male	156	16.66	Normal weight
C	15	Male	160	18.75	Normal weight
C	16	Female	79	14.19	Normal weight
C	17	Female	120	15.46	Normal weight
C	18	Female	120	17.56	Normal weight
C	19	Female	120	15.30	Normal weight
C	20	Female	122	17.20	Normal weight
C	21	Female	132	19.15	Normal weight
C	22	Female	132	18.42	Normal weight
C	23	Female	150	20.08	Normal weight
C	24	Female	158	15.96	Normal weight

Group O, patients with osteogenesis imperfecta; Group C, patients from the control group.

## Discussion

According to our knowledge, this is the first study that used bioelectrical impedance and phase angle calculation in pediatric patients with osteogenesis imperfecta as an adjunct test to assess nutritional status. The PA has been used as a marker of nutritional status in clinical practice of adults^6^^-^ 9 and children^13^ and, when associated with anthropometric data of weight and height in patients at nutritional risk, it is a good screening tool.

Nagano et al. suggested the PA as a useful parameter for nutritional assessment of body cell mass in stable pediatric patients.^13^ It has been shown to be important in the assessment of severity and prognosis, as it reflects different electrical properties of tissues that are affected by disease, nutritional status and hydration, considering it evaluates different dimensions of nutritional status, incorporating both the functional and the morphological assessment.^4^^,^ 13 ^-^ 15 Therefore, its value is influenced by body cell mass and the amount of body fluid.^16^^,^ 17

According to Barbosa-Silva et al., this parameter allows routine monitoring through a single or sequenced analysis of the sick child, which can be interpreted as an indicator of fluid distribution between the intra- and extracellular spaces and integrity of all cell membranes.^15^ The low value of PA suggests cell death or decreased integrity of cell membranes, while high values are compatible with higher value of reactance and a large amount of intact cell membranes.^14^^,^ 15

In this study, the statistically lower PA values in the OI group are probably related to lower reactance values. Although there is no statistically significant difference regarding the reactance data (shown in Table 1), there is a clear tendency toward lower values in patients with OI, probably influenced by osteocyte membrane integrity and impaired nutritional status of these children.

The anthropometric assessment of patients with OI according to the WHO criteria may not be the most appropriate as, in some cases, it was necessary to perform the measurement of height/length with the patient lying down in bed. This prevents the classification reliability and makes secondary analyses, such as the phase angle, be preferred to assess nutritional risk.

OI is a rare disease that causes the bone mineral density reduction and patients are thus susceptible to multiple fractures and consequent deformities, preventing an accurate analysis of nutritional risk. The main objectives of OI treatment are to maximize mobility and activities of daily living, as well as reduce bone pain and fragility,^18^ without neglecting nutritional status improvement, directly associated with this development. The classification of nutritional status according to the WHO criteria for BMI/A showed no significant alterations in most patients. However, as mentioned before, these values might be unreliable regarding the actual height/length of the child due to deformations, with evident limb shorting. Thus, an auxiliary analysis becomes necessary.

Barufaldi et al. also pointed out that the WHO criteria are less sensitive in relation to the ability to detect malnutrition and nutritional risk situations and more sensitive to detect overweight. Even achieving greater differentiation regarding excess weight, this criterion does not discriminate the reason for the change in relation to body composition. Thus, the use of the PA could be considered a useful and sensitive tool to classify bodily changes regarding malnutrition in patients with OI, although less specific.^19^

The bone health of patients with OI can be improved through a multidisciplinary approach, with the use of pamidronate, adequate manual therapies^20^ and nutritional therapy. All patients in this study were receiving this drug. Because bone health is associated with adequate nutrition, it is necessary to be aware of the nutritional status of these children during hospitalization and at discharge recommendations.

The improvement of bone mineral density is associated with micronutrient intake (especially of phosphorus, calcium and zinc), as well as an adequate supply of proteins with high biological value for age.^21^ Furthermore, in the case of newborns, exclusive breastfeeding during the first months of life should be encouraged, as it has been shown to be a protective factor for bone formation.^22^ Thus, the calculation of the phase angle used as an auxiliary tool to assess the nutritional status would be important, as the analysis does not depend on anthropometrical parameters, such as weight and height or length, and possibly improved the nutritional status of the patients.

This study has some limitations. The first would be the small number of patients, as it is a rare disease. The second would be to impossibility to create groups in relation to the type of osteogenesis. A third would be the fact that, proportionally, there are more female patients in the group of patients with OI when compared to the control group. We believe, however, that by reporting bioelectrical impedance values compatible with international data in patients with similar school age and adolescence phase, pubertal alterations, if present, did not influence the final value of the analysis.^4^

Despite these limitations, as there are few studies about the nutritional status and classification of children with osteogenesis imperfecta, this study is important to evaluate the use of new tools (PA) to improve the treatment of those with the condition. For more data to be collected, we suggest the use of bioelectrical impedance as a routine form of assessment in these patients, as well as the inclusion of resistance and reactance data on the National Register for osteogenesis imperfecta (Reference Center for Osteogenesis Imperfecta - Centro de Referência em Osteogênese Imperfeita - Crois - Instituto Fernandes Figueira - FIOCRUZ), a situation which would promote the analysis of a higher number of patients and the obtaining of more consistent data.

We conclude that the use of phase angle can help to classify the nutritional status of children with osteogenesis imperfecta and improve the nutritional and multidisciplinary approach. As it is a low cost procedure, it can be routinely incorporated into the care of patients with OI in our country. The prospective analysis of these patients is suggested, as well as their inclusion in the national database to facilitate the nutritional improvement of these patients, as well as of their bone health.
